# Computational Study of Hippocampal-Septal Theta Rhythm Changes Due to Beta-Amyloid-Altered Ionic Channels

**DOI:** 10.1371/journal.pone.0021579

**Published:** 2011-06-24

**Authors:** Xin Zou, Damien Coyle, KongFatt Wong-Lin, Liam Maguire

**Affiliations:** Intelligent Systems Research Centre, University of Ulster Magee Campus, Derry, Northern Ireland, United Kingdom; Nathan Kline Institute and New York University School of Medicine, United States of America

## Abstract

Electroencephagraphy (EEG) of many dementia patients has been characterized by an increase in low frequency field potential oscillations. One of the characteristics of early stage Alzheimer’s disease (AD) is an increase in theta band power (4–7 Hz). However, the mechanism(s) underlying the changes in theta oscillations are still unclear. To address this issue, we investigate the theta band power changes associated with β-Amyloid (Aβ) peptide (one of the main markers of AD) using a computational model, and by mediating the toxicity of hippocampal pyramidal neurons. We use an established biophysical hippocampal CA1-medial septum network model to evaluate four ionic channels in pyramidal neurons, which were demonstrated to be affected by Aβ. They are the L-type Ca^2+^ channel, delayed rectifying K^+^ channel, A-type fast-inactivating K^+^ channel and large-conductance Ca^2+^-activated K^+^ channel. Our simulation results demonstrate that only the Aβ inhibited A-type fast-inactivating K^+^ channel can induce an increase in hippocampo-septal theta band power, while the other channels do not affect theta rhythm. We further deduce that this increased theta band power is due to enhanced synchrony of the pyramidal neurons. Our research may elucidate potential biomarkers and therapeutics for AD. Further investigation will be helpful for better understanding of AD-induced theta rhythm abnormalities and associated cognitive deficits.

## Introduction

Alzheimer’s disease (AD) is a neurodegenerative disease associated with memory deficits and cognitive decline, which may be induced by anatomical and physiological changes in the brain. AD is characterized by two neuropathological structures: neurofibrillary tangles and senile plaques. The neurofibrillary tangles are the residue of neuronal death, which may be caused by the microtubule-binding protein, tau, becoming hyperphosphorylated. The senile plaques are mainly composed of Aβ. Aβ acts as a neurotoxin causing neuronal dysfunction and apoptosis [1]. As Aβ precedes tau protein in AD progress [2], we will focus on Aβ in this work.

It has also been found that pathological changes in the brain can lead to abnormalities in oscillations of field potentials recorded by EEG [3], [4], [5] and local field potential (LFP) [6]. The AD induced brain field potentials oscillation abnormalities and the cause of these abnormalities are complex. Previous studies have shown that early stages of AD are characterized by an increase in theta band (4–7 Hz) power and decrease in beta band (13–30 Hz) and alpha band (8–12 Hz) power [3], [7], [8]. The abnormalities may be caused by the pathological changes in many brain regions, e.g., medial temporal lobe and cortex [9]. In this work, we will focus on the Aβ affected hippocampal pyramidal neurons and the associated theta band power changes for various reasons, e.g., the hippocampus is affected at the early onset of AD [10], especially the pyramidal cells in the hippocampus [4] and the hippocampus and the associated medial septum are one of the major sources of low frequency theta oscillation.

Aβ (mainly Aβ_1-42_) can oligomerize and permeate into the cell membrane, which can break down the regulation of Ca^2+^ movement and ionic homeostasis of neurons [11]. Aβ may change the activity of various ionic channels, e.g., Aβ has been found to be able to potentiate L-type Ca^2+^ channels [12], [13]. Aβ also affects K^+^ channels, which have an intimate relationship with the cell resting potential and membrane repolarization. It has been reported in [14] that low concentration of Aβ blocks A-type fast-inactivating K^+^ channels and a high concentration of Aβ can also block delayed rectifying K^+^ channels. The effect of Aβ on large-conductance Ca^2+^-activated K^+^ channels (BK) is still a subject of debate. BK channels were reported to be activated by Aβ [15], [16], [17]. However, other research has shown that Aβ suppresses BK channels in some cases [18], [19], [20]. Arispe et al. [21] proposed a hypothesis that Aβ could also form new cation channels in neuronal membrane. In addition, Aβ can disturb the neurotransmitter systems by inducing cholinergic and glutamatergic dysfunctions [22]. All of the pathological changes outlined above may result in alterations in theta band power. As a first step in our study, we focus on the changes in these four ionic channels, i.e., L-type Ca^2+^ channel (I_Ca_); A-type fast-inactivating K^+^ channel (I_A_), delayed rectifying K^+^ channel (I_K_) and large-conductance Ca^2+^-activated K^+^ channel (I_CT_), and evaluate any corresponding change in hippocampal theta band power.

To investigate the effect of Aβ on hippocampo-septal theta rhythm, we make use of a biophysical model of the hippocampal CA1 region and the medial septum. The spiking neuronal network model consists of hippocampal principle pyramidal neurons, basket and OLM interneurons and the medial septal MSGABA neurons. The model of pyramidal neurons is constructed based on [23], [24]. The basket, OLM and MSGABA interneurons are modelled in the same way as presented in [25], [26]. Synapses in our work are mediated by typical neurotransmitters GABA_A_, NMDA and AMPA, which are based on [27]. The aim is to evaluate the relationship between Aβ induced changes in ionic channels (I_Ca_, I_K_, I_A_ and I_CT_) and the theta band power alterations. The effects of Aβ on those channels are simulated by changing the amplitudes of these ionic currents. Our simulation results show that theta band power is highly dependent on I_A_ but not I_Ca_, I_K_ and I_CT_. In particular, theta band power significantly increases with a decrease in I_A_. We propose that this increased theta band power is induced by the enhanced synchrony of pyramidal neurons. This hypothesis is supported by our simulation results.

## Methods

**Figure 1:**
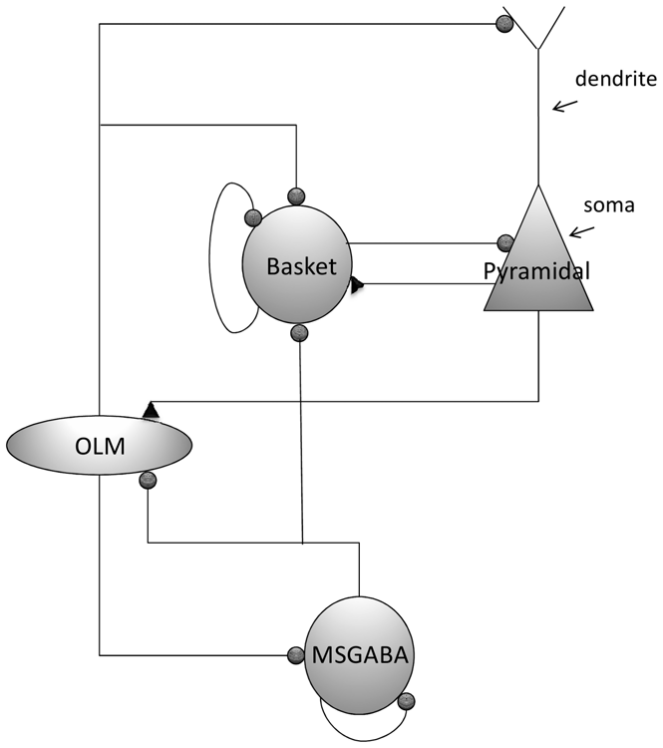
Hippocampo-septal network architecture. The network consists of four types of neuronal populations, i.e., pyramidal, basket, OLM and MSGABA neurons. Inhibitory GABA_A_-mediated synaptic connections are indicated by ‘•’, and excitatory AMPA and NMDA-mediated synaptic connections are indicated by ‘▴’.

We construct a network model of hippocampus CA1-medial septum based on the Hodgkin-Huxley type formalisms presented in [27]. The model incorporates three types of neurons from the hippocampus, i.e., excitatory pyramidal, inhibitory basket and OLM neurons and inhibitory MSGABA neurons from the medial septum. These neurons have been demonstrated to contribute to theta rhythm activity in *in vivo* experiments [28], [29], [30] and in simulation studies [26], [31]. A schematic diagram of the neuronal network architecture is illustrated in Figure 1. Each type of neuron in Figure 1 represents a population of identical neurons.

The pyramidal neurons are modelled by a two-compartmental model, one for the soma and the other for the dendrite. As in [23], the soma compartment has spike generating currents I_Na_ and I_K_ and the dendrite contains a calcium dependent potassium current I_AHP_. Both the soma and dendrite contain leakage currents I_L_ and high-threshold L-type calcium currents I_Ca_ plus hyperpolarization-activated currents 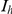. The pyramidal neurons in hippocampus CA1 contain additional ionic currents to account for different neuronal functions [24]. In this work, we select some of these currents, which have been shown to be affected by Aβ. As a result, our model also contains an A-type potassium current I_A_ and a large-conductance calcium dependent potassium current I_CT_ in the soma and dendrite, respectively. The Hodgkin-Huxley type dynamical equations for the pyramidal neurons are:
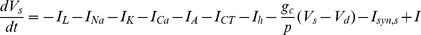
(1)




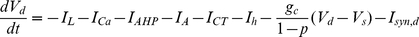
(2)where subscript *s* and *d* denotes soma and dendrite, respectively. *I* is the injected DC current and *I_syn_* is the synaptic currents from interneurons.

The other three inhibitory neurons are modelled as one-compartment. The model of basket neurons has I_Na_, I_K_, and leakage current I_L_, Eq. 3. The model of OLM has I_Na_, I_K_, I_L_, I_Ca_, hyperpolarization activated current I_h_ and I_AHP_, Eq. 4. MSGABA contains I_Na_, I_K_, I_L_ and a slowly inactivating potassium current I_KS_, Eq. 5.
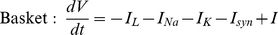
(3)

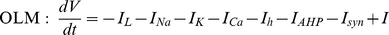
(4)

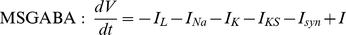
(5)


To emulate heterogeneity in the real brain tissues, the injected DC current *I* for each neuron is not chosen to be identical. This is done by allowing *I* to follow a Gaussian distribution with mean 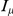 and standard derivation 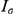. 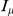 for the pyramidal, basket, OLM and MSGABA neuronal populations are chosen to be 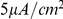, 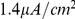, 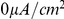 and 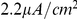, respectively. As there is no agreement on the specific 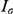 to be used, we chose 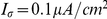 for all populations for simplicity. This heterogeneity will be implemented in all our simulations. Definitions of all the other parameters are given in Appendix S1.

The number of pyramidal, basket, OLM and MSGABA neurons are 10, 100, 30 and 50, respectively [27]. In the network, the pyramidal neurons innervate basket neurons via neurotransmitter AMPA and OLM via AMPA+NMDA, other synaptic connections are mediated by GABA_A_ neurotransmitter. We model their effects with rise and decay time constants of their synaptic gating variables. It has been shown in [32] that the synaptic time constants have the equivalent effect of the conduction delays on the postsynaptic activities. Slight changes in these time contents do not affect our conclusion. The network is constructed using a sparse connectivity i.e., the neurons are randomly coupled with a fixed average number of pre-synaptic inputs/post-synaptic outputs per neuron. The number of pre-synaptic inputs/post-synaptic outputs is adjusted according to [27].

We compute the LFP signal as a sum of the values of the synaptic currents of the pyramidal neurons [33]. This is under the assumption that pyramidal neurons contribute more to the overall signal due to their approximate open field arrangement. The fast components of the LFP are reduced by low pass filtering (0–40 Hz). The power spectrum is obtained by a fast Fourier transform with a 2 s length Hanning window. The relative theta band power (% of the total power) is calculated. A membrane potential noise that follows a Gaussian distribution with zero mean and *1.5mV* standard derivation is also introduced in some of the following simulations. The membrane noise is randomly generated in each trial that lasts for 10 s. Each presented result is obtained from simulations averaged over 15 trials of the model representing different individual patients. We found that higher number of trials does not alter the obtained average theta band power. The results from trials of the model run with normal parameter settings are considered as a “healthy” control group whereas trials with alterations to parameters of various ionic channels simulate deficiencies and are considered as potential different “patient” groups. Various ionic channels are potentiated or suppressed to simulate the effects of Aβ, which will be presented in the next section. All of the results were obtained by adjusting the ionic currents in the pyramidal neurons only. The statistical significance of the differences between groups is evaluated using a one-way ANOVA test. Error bars are standard errors.

## Results

**Figure 2:**
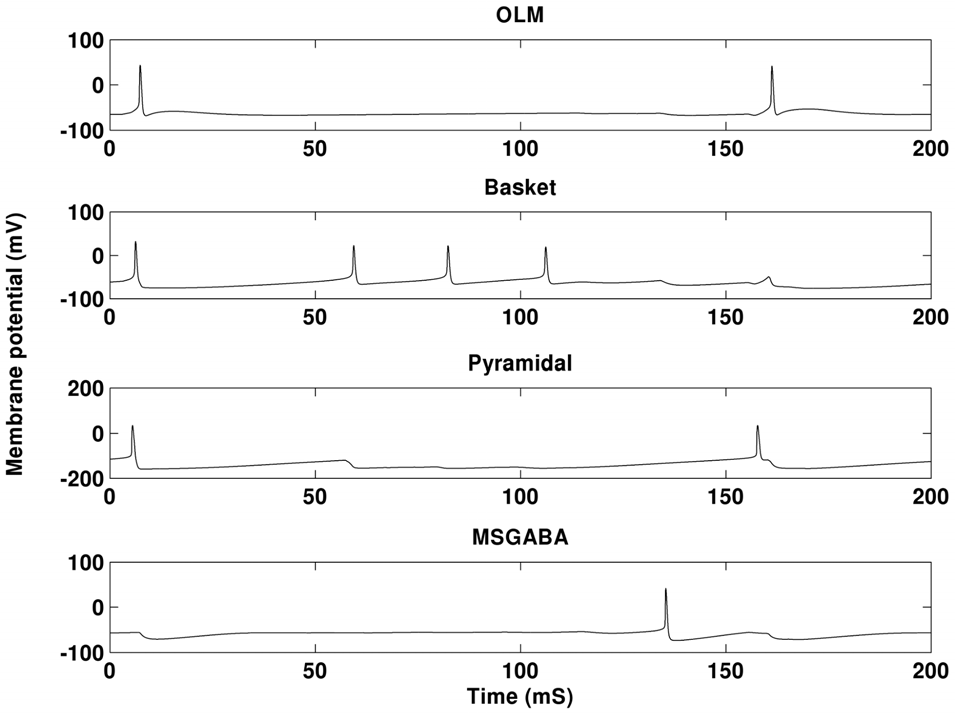
Membrane potential dynamics in theta oscillation. Each individual network theta oscillation period consists of spikes of different neuronal populations clustering around different phases. The figures are obtained in control condition without membrane noise.

The dynamics of neurons in theta oscillation obtained in control condition are demonstrated in Figure 2. To better illustrate the spiking phases of different neuronal populations, membrane noise is removed. Figure 2 shows that theta oscillation is generated by the spiking of different neuronal populations clustered at certain phases. Assuming a network theta oscillation begins with spikes from the pyramidal neurons. Then the OLM neurons are evoked via the excitatory synaptic connections from the pyramidal neurons, which produce a feedback inhibition to the pyramidal neurons. The basket neurons then gradually depolarize and produce series of spikes. The spikes of basket neurons are inhibited by the spiking of MSGABA neurons. The slowly inactivating potassium current I_KS_ in MSGABA neuron plays a very important role in the theta generation, which is referred to as a ‘pacemaker’ for theta rhythm [26].

**Figure 3:**
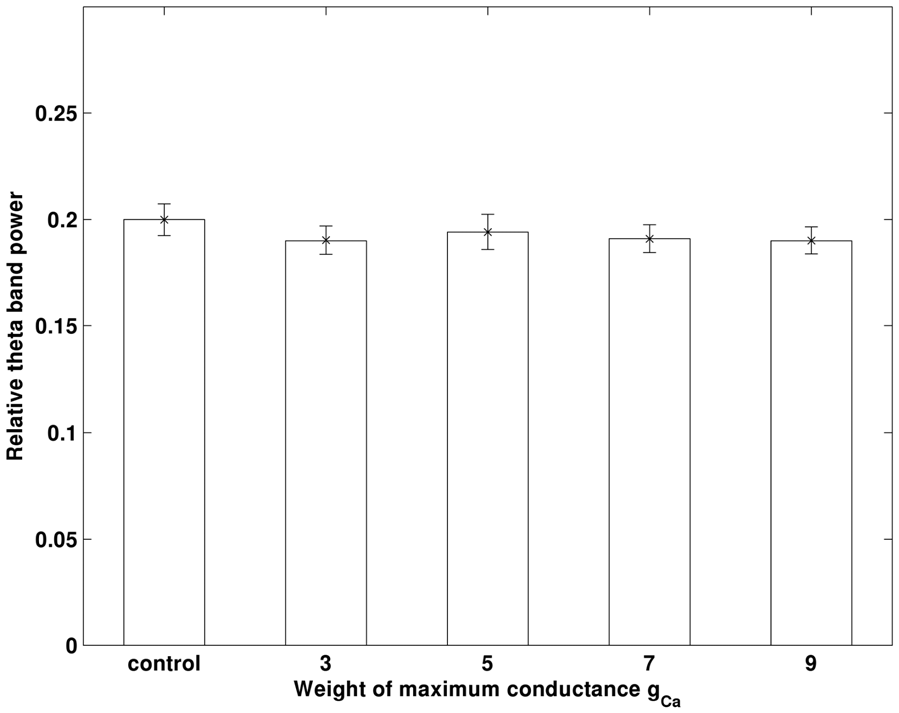
Increase in g_Ca_ does not induce changes in theta band power. In the figure, the obtained average theta band power of each experiment is illustrated. Errorbar is standard error.

It has been pointed out that the main cause of the loss of intracellular calcium homeostasis in AD patients is that Aβ can potentiate the L-type Ca^2+^ channels (I_Ca_) [13], which causes a large influx of Ca^2+^ into the cells. The mechanism of Aβ increasing the influx of Ca^2+^ is still unclear. Aβ may form new cation channels and/or alter the existing L-type Ca^2+^ channels. In our simulations, we emulate the effect of Aβ by increasing the maximum conductance of the L-type Ca^2+^ channels. The obtained theta band power with enhanced I_Ca_ is presented in Figure 3. It can be seen that changes in L-type Ca^2+^ channels do not cause a change in theta rhythm.

**Figure 4:**
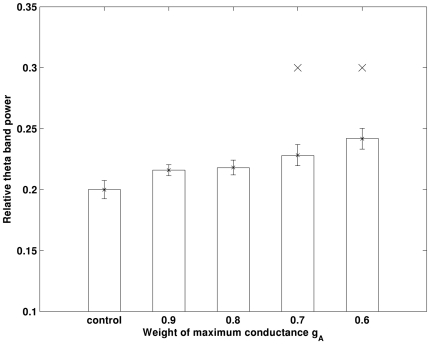
Theta band power increases with decrease in g_A_. × indicates that power is significantly larger than that obtained in control condition (*p<0.05*). Errorbar is standard error.

**Figure 5:**
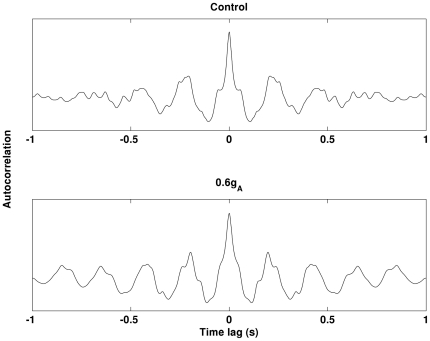
The auto-correlations of a summation of membrane potentials obtained in control and 0.6g_A_ conditions. Theta rhythm is strengthened by decreased g_A_. Both of the results are obtained in the same noisy and heterogenous condition obtained in single trial.

**Figure 6:**
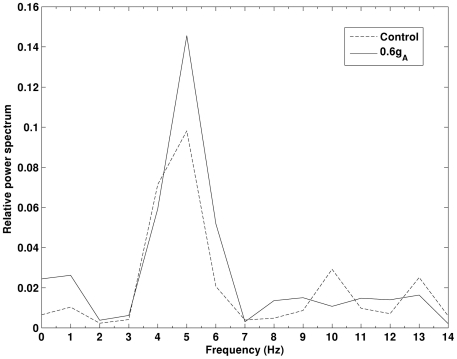
More significant power spectrum peak in theta band in 0.6g_A_ condition than in control condition. Both of the results are obtained in the same noisy and heterogenous condition obtained in single trial.

**Figure 7:**
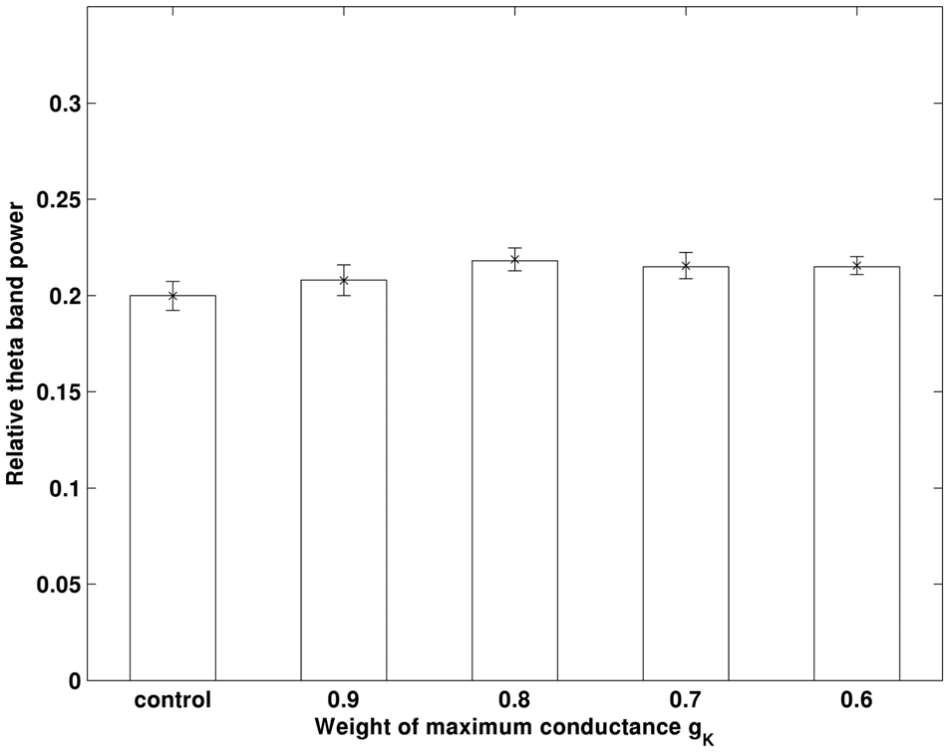
Decrease in g_K_ does not induce significant changes in theta band power. Errorbar is standard error.

Aβ also blocks some K^+^ ionic channels in pyramidal neurons, e.g., I_A_ and I_K_ [14], [34]. The experimental results showed that Aβ is more likely to block the channel from outside the neurons. Therefore we emulate the effect of Aβ by decreasing the maximum conductance of I_A_ and I_K_, respectively. Furthermore, it has been shown that I_A_ has larger density in dendrite compared with soma [35] and Aβ have much greater effect on the dendrite I_A_ [36], [37]. Based on these findings, only I_A_ in the dendrite will be reduced. The simulation results obtained in control and decreased I_A_ in the dendrite only conditions are illustrated in Figure 4. Our simulation shows that theta band power is significantly increased (*p<0.05*) as I_A_ decreases. An example of the auto-correlation of the summation of all membrane potentials and the corresponding band power in control and 0.6g_A_ conditions is illustrated in Figure 5 and 6. It can be seen that theta oscillation and its power is significantly increased with low g_A_. Similar changes in theta band power due to I_K_ (via g_K_) are not observed, as illustrated in Figure 7.

**Figure 8:**
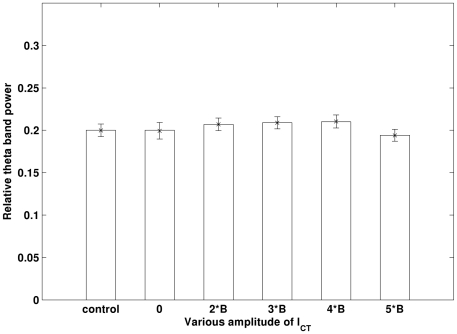
Change in I_CT_ does not induce significant changes in theta band power. Both the completely blocked I_CT_ (0) and the potentiated I_CT_ (2B, 3B, 4B, 5B) are evaluated. Errorbar is standard error.

As AD disturbs the homeostasis of Ca^2+^, the Ca^2+^-activited BK channel (I_CT_) is vulnerable to AD pathology. BK channel can adjust the spike broadening during repetitive firing [38] and spiking frequency [39]. Previous research reveals that the activity of BK channel is probably promoted by Aβ [17]. However, other research reports that BK channel is suppressed in some cases [18], [19], [20]. Therefore, we have simulated both increased and blocked I_CT_ in our simulations. I_CT_ is potentiated by increasing the fraction of Ca^2+^ influx, *B* (see Appendix S1). The simulation results are illustrated in Figure 8. It can be seen that neither blockage nor potentiation in I_CT_ can affect theta rhythm.

The simulation results have shown that a decrease in I_A_ can significantly increase theta band power. To evaluate whether this is due to an enhanced synchrony of neuronal populations, we calculate the population coherence coefficient [40]. In this section, g_A_ is decreased in both soma and dendrite simultaneously. The long time interval *T* (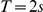 in our experiment) is first divided into small bins of 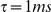 and spike trains of the *i* ^th^ and *j* ^th^ neurons in the population are given 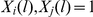
or 0, 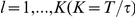, where ‘*1*’ denotes spiking and ‘*0*’ resting. The coherence coefficient 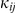 between the trains can be calculated as
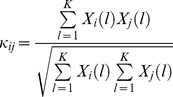
(6)


**Figure 9:**
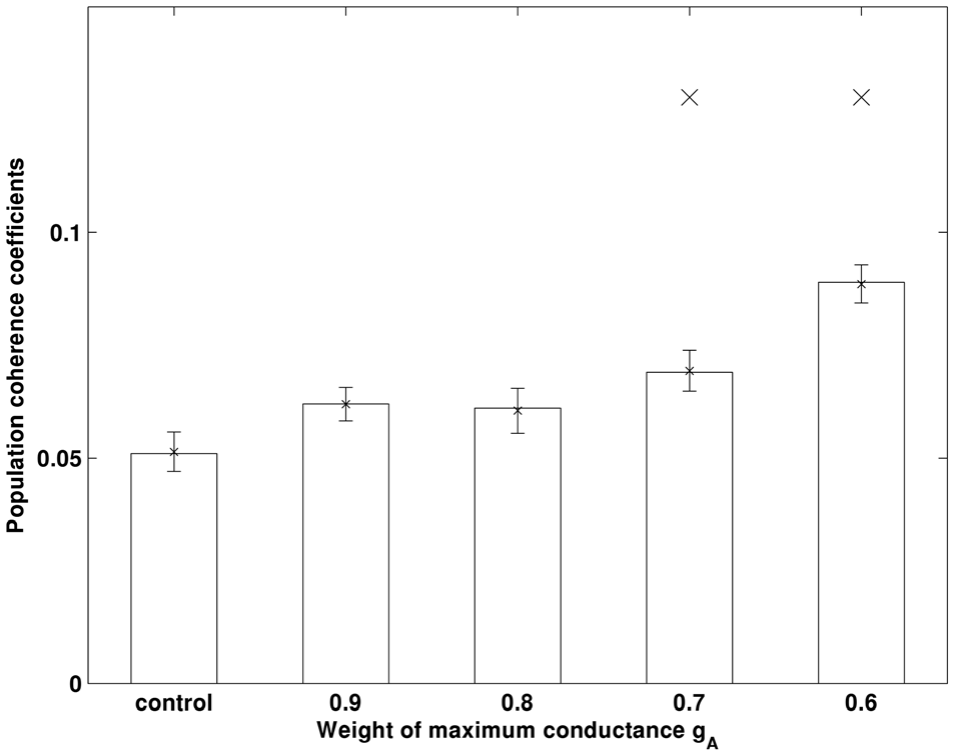
The pyramidal neuronal population coherence coefficients increase with *g_A_* decreases. ‘×’ indicates the coherence coefficient is significantly larger than that obtained in control condition (*p<0.01*). Errorbar is standard error.

The whole population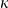 is obtained by averaging all of the combinations of *i* and *j*. 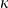 is calculated for the control group and the group with decreased g_A_. In the following simulations, I_A_ in both soma and dendrite are decreased simultaneously. The obtained 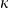 is illustrated in Figure 9. Consistent with our hypothesis, population synchrony is significantly increased as g_A_ decreases (*p<0.001*).

**Figure 10:**
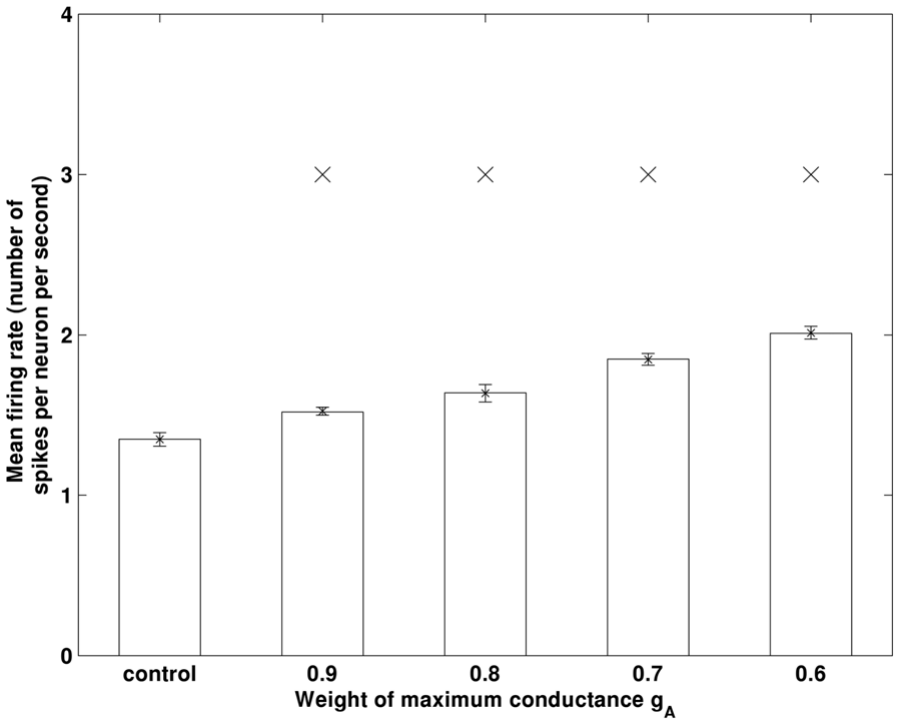
The pyramidal neuronal population firing rates increase with decrease in g_A_. ‘×’ indicates the firing rate is significantly larger than that obtained in control condition (*p<0.001*). Errorbar is standard error.

The increased synchrony is probably caused by the enhanced excitability and firing rate of the pyramidal neurons. To support this hypothesis, the firing rates of the pyramidal neuronal population with various values of g_A_ are shown in Figure 10. It can be clearly seen that the decreased g_A_ has enhanced the excitability of the pyramidal neurons and their firing rates. Therefore, we suggest that when I_A_ is decreased, the pyramidal neurons become more excitable. During the peak of each pyramidal population theta cycle, more pyramidal neurons spike simultaneously, which enhances the synchrony of the population.

In summary, our simulations have shown that a decrease in I_A_ in the pyramidal neurons induces an increase in theta band power by recruiting more pyramidal neurons to fire.

## Discussion

AD is usually accompanied with alterations in neuronal network oscillations. The patterns of oscillation changes in different frequency bands have been used to discriminate the AD-induced dementia from the other dementias [41]. The aim of our work is to better understand the mechanisms underlying these oscillation abnormalities. We have investigated rhythms using other types of models and looked at connectivity changes in our previous work, e.g., we have investigated the AD-induced alpha rhythm abnormalities [42] and the relationship between changes in alpha and theta rhythms [43] using an abstract model. In this work, we investigated the Aβ-induced theta oscillation abnormality based on a conductance-based hippocampo-septal model.

Previous experimental results have demonstrated that Aβ can induce neuronal dysfunction by altering certain ionic channels. The computational simulations have shown that some of the ionic channels play critical roles in the neuronal network oscillations, e.g., [26]. However, the mechanisms underlying Aβ induced hippocampo-septal theta rhythm alteration remains unclear. In this work, the change in theta band power caused by Aβ has been investigated using a conductance-based hippocampus CA1 and medial septum network model. Based on previous experimental results, the effect of Aβ was emulated by blocking or potentiating specific ionic channels. Then the corresponding theta band power was calculated and compared with that obtained in a control (normal) condition. We have evaluated four types of ionic channels, one Ca^2+^ and three K^+^ channels. We have identified that only a decrease in fast-inactivating K^+^ currents (I_A_) affected theta band power. To explain its mechanism, we have proposed that the blockage of I_A_ by Aβ increases the excitability of pyramidal neurons, which led to more synchrony of pyramidal neuronal firings. The synchronized firing state then propagated to other neuronal populations. As a result, theta band power was increased. Our hypothesis has been supported by various simulations. Our computational work has shown that Aβ-induced I_A_ depression could be an important factor in causing theta rhythm abnormalities in AD. Our results may have implications for the development of the AD biomarkers and therapeutics. For example, drugs which can potentiate I_A_ may be used to counteract the affect of Aβ. In fact, cannabinoids which can potentiate I_A_ [44], have been successfully used in AD treatment [45].

**Figure 11:**
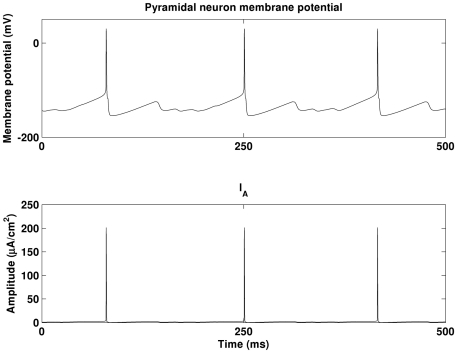
An example of dynamics of a pyramidal neurons and the associated brief transient I_A_.

In this work, we have observed that decreased I_A_ can enhance the excitability of the pyramidal neurons and result in higher theta band power. However, how alterations in I_A_ change the excitability of pyramidal neurons is still unclear. If the activation of I_A_ is long lasting, then the mechanism may be straightforward, as reducing a long lasting current may allow more neurons to spike per theta cycle. Figure 11 illustrates an example of dynamics of a pyramidal neuron and associated I_A_. As shown, I_A_ actually operates very briefly as compared to theta rhythm. It resets shortly after a spike. Therefore, the mechanism underlying I_A_-induced firing rate changes is a topic which deserves further attention and is the focus of our on-going research. Furthermore, we recognize that not all experimental observations fit with this picture of enhanced theta band power. For example, in [46], theta band power was found to decrease in rats’ hippocampus injected with Aβ. The mechanism underlying Aβ induced theta band power decrease is also currently being investigated.

The long term potentiation and depression of synapses in the hippocampus play critical roles in the formation and processing of memories. Previous research [27] has shown that synaptic changes can also induce alterations in theta band power. To achieve this, the afference from other parts of brain, e.g., acetylcholine neuromodulation from medial septum, may be incorporated into the model. Furthermore, it has been found that AD is usually associated with an increase chance of unprovoked epilepsy [47]. In a recent study [48], it has been shown that Aβ could be the main cause of epilepsy in AD due to hippocampal network hyper-excitability. Our work could provide a potential explanation for this observation. We will address these issues in our future work.

## Supporting Information

Appendix S1 **Definition of the model parameters.**
(DOC)
