# Supplementary material for: Computational Study of Hippocampal-Septal Theta Rhythm Changes Due to Beta-Amyloid-Altered Ionic Channels
Source: PLoS One. 2011 Jun 24;6(6):e21579. doi: 10.1371/journal.pone.0021579 (PMC3123375; doi:10.1371/journal.pone.0021579)
Supplement: Appendix S1 — Definition of the model parameters. (DOC) [file pone.0021579.s001.doc]

Appendix S1

The membrane capacitance $C=1\mu F/cm^{2}$ for all of the follow equations, therefore it will be ignored. The τ in (*ms*); *E* and *V* in (*mV*); *I* in ($\mu A/cm^{2}$); g in ($mS/cm^{2}$);$\alpha$ and $\beta$ in (*ms^-1^*); *K* in ($\mu M$); B in ($\mu M(ms\mu A)^{-1}cm^{2}$) and the rest are dimensionless constant. We used an Euler method for numerically integrating the stochastic differential equations, using a time step of 0.01ms. Smaller time steps do not change our results.

A.1 Neuronal dynamics

The pyramidal somatic and dendritic membrane potentials, denoted by V_s_ and V_d_, obtains the following equations:

$\frac{dV_{s}}{dt}=-I_{L}-I_{Na}-I_{K}-I_{Ca}-I_{A}-I_{CT}-I_{h}-\frac{g_{c}}{p}(V_{s}-V_{d})-I_{syn,s}+I$ (A1)

$\frac{dV_{d}}{dt}=-I_{L}-I_{Ca}-I_{AHP}-I_{A}-I_{CT}-I_{h}-\frac{g_{c}}{1-p}(V_{d}-V_{s})-I_{syn,d}$ (A2)

Where $g_{c}=2mS/cm^{2}$ is the coupling conductance between soma and dendrite, *p=somatic area/total area=0.5*. *I* is the injected DC current and *I_syn_* is the synaptic currents. *I_L_=g_L_(V-E_L_)*. In our work, all of the ionic currents are modelled by the Hodgkin-Huxley type formalism, thus the dynamic of a gating variable *x* satisfies first-order kinetics,

$\frac{dx}{dt}=\varphi_{x}[\alpha_{x}(1-x)-\beta_{x}x]=\varphi_{x}[x_{\infty}-x]/\tau_{x}$ (A3)

This equation will be used to calculate all of the gating variables.

| Channel | Definition | Parameters |
| --- | --- | --- |
| *I_Na_* | $g_{Na}m_{\infty}^{3}h(V-E_{Na})$ | $m_{\infty}=\alpha_{m}/(\alpha_{m}+\beta_{m})$ $\alpha_{m}=-0.1(V+33)/\exp[-0.1(V+33)-1]$ $\beta_{m}=4\exp[-(V+58)/12]$ $\alpha_{h}=0.07\exp[-(V+50)/10]$ $\beta_{h}=1/\exp[-0.1(V+20)+1]$ |
| *I_K_* | $g_{K}n^{4}(V-E_{K})$ | $\alpha_{n}=-0.01(V+34)/\exp[-0.1(V+34)-1]$ $\beta_{n}=0.125\exp[-(V+44)/25]$ |
| *I_Ca_* | $g_{Ca}m_{\infty}(V-E_{Ca})$ | $m_{\infty}=1/\exp[-(V+20)/9]$ |
| *I_AHP_* | $\begin{matrix} g_{AHP}[Ca^{2+}]/([Ca^{2+}]+K_{D}) \\ (V-E_{K}) \end{matrix}$ | $\frac{d[Ca^{2+}]}{dt}=-[Ca^{2+}]/\tau_{Ca}-BI_{Ca}$ $\tau_{Ca}=1000,B=0.002,K_{D}=30\mu M$ |
| *I_A_* | $g_{A}a^{3}b(V-E_{K})$ | $\alpha_{a}=-0.05(V+20)/\{\exp[-(V+20)/15]-1\}$ $\beta_{a}=0.1(V+10)/\{\exp[(V+10)/8]-1\}$ $\alpha_{b}=0.00015/\exp[(V+18)/15]$ $\beta_{b}=0.06/\{\exp[-(V+73)/12]+1\}$ |
| *I_CT_* | $g_{CT}c^{2}d(V-E_{K})$ | $\begin{matrix} \alpha_{c}=-0.0077(V+V_{shift}+103)/\{\exp[-(V+V_{shift}+103)/12] \\ -1\} \end{matrix}$ $\beta_{c}=0.91-\alpha_{c}$ $\alpha_{d}=1/\exp[(V+79)/10]$ $\beta_{d}=4/\{\exp[-(V-82)/27]+1\}$ $V_{shift}=40\log([Ca^{2+}]/13.805)$ $\tau_{Ca}=0.9,B=0.06$ |
| $I_{h}$ | $g_{h}H(V-E_{h})$ | Values of $H_{\infty}$and $\tau_{H}$ corresponding to different *V* can be found in (supplementary) |

The values of the other parameters are $\varphi=4$, *g_L_=0.1* and *g_Ca_=0.5* for soma and dendrite, *g_Na_=45, g_K_=18, g_A_=30* *g_CT_=140* and *g_h_=0.01* for soma and *g_AHP_=5, g_A_=60* *g_CT_=70* and *g_h_=0.02* for dendrite; *E_L_=-65, E_Na_=55, E_K_=-80, E_Ca_=120,* and $I_{\mu}=5.5$.

The OLM neuron is described as a single compartment model,

(A4)

| Channel | Definition | Parameters |
| --- | --- | --- |
| *I_Na_* | $g_{Na}m_{\infty}^{3}h(V-E_{Na})$ | $\alpha_{m}=-0.1(V+35)/\exp[-0.1(V+35)-1]$ $\beta_{m}=4\exp[-(V+60)/18]$ $\alpha_{h}=0.07\exp[-(V+58)/20]$ $\beta_{h}=1/\exp[-0.1(V+28)+1]$ |
| *I_K_* | $g_{K}n^{4}(V-E_{K})$ | $\alpha_{n}=-0.01(V+34)/\exp[-0.1(V+34)-1]$ $\beta_{n}=0.125\exp[-(V+44)/80]$ |
| *I_Ca_* | $g_{Ca}m_{\infty}^{2}(V-E_{Ca})$ | $m_{\infty}=1/\{\exp[-(V+20)/9]+1\}$ |
| *I_AHP_* | $\begin{matrix} g_{AHP}[Ca^{2+}]/([Ca^{2+}]+K_{D}) \\ (V-E_{K}) \end{matrix}$ | $\frac{d[Ca^{2+}]}{dt}=-[Ca^{2+}]/\tau_{Ca}-BI_{Ca}$ $\tau_{Ca}=80,B=0.002,K_{D}=30\mu M$ |
| *I_h_* | $g_{h}H(V-E_{h})$ | $H_{\infty}=1/\{\exp[(V+80)/10]+1\}$ $\begin{matrix} \tau_{H}=200/\{\exp[(V+70)/20]\}+\exp[-(V+70)/20] \\ +5\} \end{matrix}$ |

The other parameters are $\varphi=5$, $g_{L}=0.1$, $g_{Na}=35$, $g_{K}=9$, $g_{AHP}=10$, $g_{Ca}=1$, $g_{h}=0.15$; $E_{L}=-65$, $E_{Na}=55$, $E_{K}=-90$, $E_{Ca}=120$, $E_{h}=-40$,$I_{\mu}=0$.

The basket neuron is described as a single compartment model,

$\frac{dV}{dt}=-I_{L}-I_{Na}-I_{K}-I_{syn}+I$ (A6)

The parameters for calculation of the ionic currents are the same as that of OLM and $I_{\mu}=1.4$.

The MSGABA neuron is described as a single compartment model,

$\frac{dV}{dt}=-I_{L}-I_{Na}-I_{K}-I_{KS}-I_{syn}+I$ (A7)

| Channel | Definition | Parameters |
| --- | --- | --- |
| I_Na_ | $g_{Na}m_{\infty}^{3}h(V-E_{Na})$ | $\alpha_{m}=-0.1(V+33)/\exp[-0.1(V+33)-1]$ $\beta_{m}=4\exp[-(V+58)/18]$ $\alpha_{h}=0.07\exp[-(V+51)/10]$ $\beta_{h}=1/\exp[-0.1(V+21)+1]$ |
| I_K_ | $g_{K}n^{4}(V-E_{K})$ | $\alpha_{n}=-0.01(V+38)/\exp[-0.1(V+38)-1]$ $\beta_{n}=0.125\exp[-(V+48)/80]$ |
| 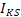 | $g_{KS}pq(V-E_{K})$ | $p_{\infty}=1/\{\exp[-(V+34)/6.5]+1\}$ $\tau_{p}=6$ $q_{\infty}=1/\{\exp[(V+65)/6.6]+1\}$ $\tau_{q}=\tau_{q0}(1+1/\{\exp[-(V+50)/6.8]+1\})$ $\tau_{q0}=100$ |

The other parameters are $\varphi=5$, $g_{L}=0.1$, $g_{Na}=50$, $g_{K}=8$, $g_{KS}=12$; $E_{L}=-50$, $E_{Na}=55$, $E_{K}=-85$, $I_{\mu}=2.2$.

A.2 Synaptic connection definition

There are three types of synaptic neurotransmitters, the inhibitory GABA_A_, the excitatory NMDA and AMPA. The GABA_A_ inhibitory post synaptic current (IPSC) is described as $I_{GABA_{A}}=g_{syn}s(V-E_{GABA_{A}})$, where the activation variable *s* is calculated by $ds/dt=\alpha F(V_{pre})(1-s)-\beta s$. The $V_{pre}$is the presynaptic neuron membrane potential, $F(V_{pre})=1/[1+\exp(-V_{pre}/K)]$. Parameters for different neurons couples are:

| basket-pyramidal (b-p) | $\alpha=10$, $\beta=0.1$, $K=2$, $E_{GABA_{A}}=-80$, $g_{syn}=5.44$ |
| --- | --- |
| OLM-basket (o-b) | $\alpha=20$, $\beta=0.1$, $K=2$, $E_{GABA_{A}}=-80$, $g_{syn}=1.76$ |
| OLM-pyramidal (o-p) | $\alpha=20$, $\beta=0.1$, $K=2$, $E_{GABA_{A}}=-85$, $g_{syn}=1.76$ |
| OLM-MSGABA (o-m) | $\alpha=20$, $\beta=0.1$, $K=0.5$, $E_{GABA_{A}}=-80$, $g_{syn}=0.5$ |
| basket-basket (b-b) | $\alpha=10$, $\beta=0.1$, $K=2$, $E_{GABA_{A}}=-75$, $g_{syn}=0.125$ |
| MSGABA-OLM (m-o) | $\alpha=10$, $\beta=0.1$, $K=2$, $E_{GABA_{A}}=-75$, $g_{syn}=0.5$ |
| MSGABA-MSGABA (m-m) | $\alpha=10$, $\beta=0.1$, $K=2$, $E_{GABA_{A}}=-75$, $g_{syn}=0.25$ |
| MSGABA-basket (m-b) | $\alpha=10$, $\beta=0.1$, $K=2$, $E_{GABA_{A}}=-75$, $g_{syn}=1$ |

The AMPA and NMDA excitatory post synaptic current (EPSP) are described as $I_{AMPA}=g_{AMPA}s(V-E_{AMPA})$ and $I_{NMDA}=g_{NMDA}B(V)s(V-E_{NMDA})$, respectively. *s* is updated as $ds/dt=\alpha[T](1-s)-\beta s$, $[T]=T_{\max}/[1+\exp(-V_{pre}+V_{p})/K_{p}]$, *V_p_=2*, *V_K_=5*, *B(V)* is calculated by $B(V)=1/\{1+\exp(-0.062V)[Mg^{2+}]/3.5\}$, where *[Mg^2+^]=1mM* by default. The $\alpha$ and $\beta$ for AMPA and NMDA are $\alpha=1.1,\beta=0.19$ and$\alpha=0.072,\beta=0.0066$, respectively. $E_{AMPA}=E_{NMDA}=0$, $g_{AMPA}=0.9$and$g_{NMDA}=0.45$. The summated synaptic current is normalized by the number of presynaptic neurons.
